# Chromosome-level genome assembly and annotation of the social amoeba *Dictyostelium firmibasis*

**DOI:** 10.1038/s41597-024-03513-8

**Published:** 2024-06-22

**Authors:** Bart Edelbroek, Jonas Kjellin, Jon Jerlström-Hultqvist, Sanna Koskiniemi, Fredrik Söderbom

**Affiliations:** Department of Cell and Molecular Biology, BMC, Uppsala University, SE-751 24 Uppsala, Sweden

**Keywords:** Genome, Microbiology, Evolutionary genetics, Data acquisition

## Abstract

*Dicytostelium firmibasis* is a member of Dictyostelia, a group of social amoebae that upon starvation display aggregative multicellularity where the amoebae transition from uni- to multicellular life. The *D. firmibasis* genome assembly that is currently available is of limited use due to its low contiguity, large number of undetermined bases, and lack of annotations. Here we used Nanopore long read sequencing, complemented with Illumina sequencing, and developmental transcriptomics as well as small RNA-sequencing, to present a new, fully annotated, chromosome-level *D. firmibasis* genome assembly. The new assembly contains no undetermined bases, and consists mainly of six large contigs representing the chromosomes, as well as a complete mitochondrial genome. This new genome assembly will be a valuable tool, allowing comprehensive comparison to *Dictyostelium discoideum*, the dictyostelid genetically tractable model. Further, the new genome will be important for studies of evolutionary processes governing the transition from unicellular to multicellular organisms and will aid in the sequencing and annotation of other dictyostelids genomes, many of which are currently of poor quality.

## Background & Summary

*Dictyostelium firmibasis* is a member of Dictyostelia, a phylogenetic group of dictyostelid social amoebae^1^. These organisms are unicellular and free-living while food is plentiful, but at the onset of starvation, about 100,000 amoebae can stream together to start a multicellular developmental program. The cells differentiate and go through distinct morphological stages, culminating in a fruiting body where 20% of the cells sacrifice themselves to form a stalk that elevates the remaining cells, which sporulate. The spores are resistant to environmental stress and await dispersal to more favorable places^2–4^.

Dictyostelids have proven to be excellent models due to their peculiar lifecycle, providing insight into the evolution of multicellularity and altruism^2,5^. Furthermore, one of the dictyostelids, the genetically tractable organism *Dictyostelium discoideum*, is used as a model for bacterial infections and to uncover the molecular mechanisms behind biological processes such as chemotaxis and phagocytosis^6–9^. In addition, the function and evolution of non-coding (nc)RNAs, such as micro(mi)RNAs, have been studied in Dictyostelia and is a main focus of our research^10–15^. Since small RNAs, such as miRNAs, are short (commonly 21 nt) and at least in *D. discoideum*, derived from AT-rich, hard to sequence intergenic regions, high quality complete genomes are essential for ncRNA studies. *Dictyostelium firmibasis* is of particular interest because it is closely related to *D. discoideum*, which has been extensively studied over the last century and was among the first protists with a fully sequenced and annotated genome^4,16^. A rough *D. firmibasis* genome assembly was available already 2012^17^, and has been valuable for comparison with *D. discoideum* or other dictyostelids to study evolution^10,18–21^. The assembly however lacks annotations, is fragmented, and contains many unresolved gaps, which limits to what extent *D. firmibasis* can be studied. Instead, a high-qualitative chromosome-level reference genome with gene annotations would give a more complete understanding of this organism and enable comprehensive comparison to other dictyostelids.

One of the challenges with sequencing *D. firmibasis*, *D. discoideum* or other closely related social amoebae is that they are particularly AT-rich, especially in intergenic regions^18,22^. This is illustrated by the *D. discoideum* genome, which has an AT-content of 86.2% in intergenic regions^19^. Not only do these repetitive stretches of adenine and thymine hinder resolving the bases during sequencing, it also complicates assembly^16^. However, advances in long-read sequencing (third-generation sequencing) have made it feasible to get high coverage of the genome, including intergenic regions, to allow for a more complete assembly^23,24^.

**Fig. 1:**
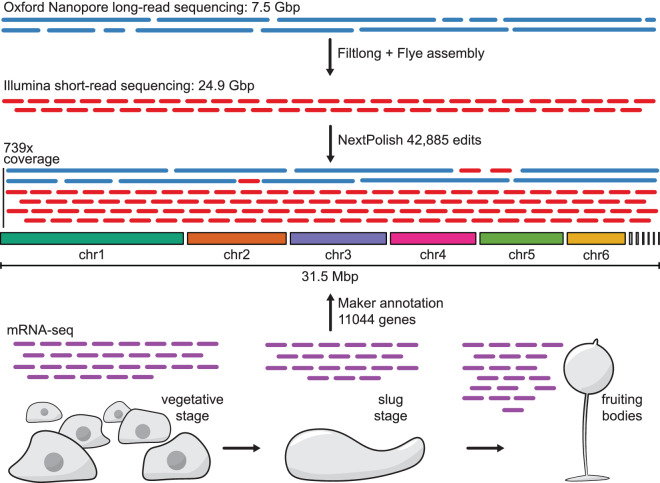
Overview of *D. firmibasis* whole genome sequencing and annotation project. De-novo assembly of *D. firmibasis* genome from 739x long- and short-read coverage resulted in 31.5 Mbp chromosome-level genome assembly, consisting of 12 contigs. mRNA-seq was performed from three distinct morphological stages (vegetative stage, slug stage, fruiting bodies) to capture also temporally expressed transcripts. For details, see Methods.

In this study, we sequenced the genome of the *D. firmibasis* TNS-C-14 strain^25^. We acquired 7.5 Gbp of Oxford Nanopore long-read sequences and 24.9 Gbp of Illumina short-read sequences and were able to de-novo assemble the *D. firmibasis* genome. This resulted in six main contigs of 9.4 Mbp to 3.9 Mbp and six small contigs of 118 kbp to 24 kbp, for a combined assembly size of 31.5 Mbp with 739 times total coverage (Fig. 1). Analysis of the telomeres and comparison to the *D. discoideum* genome confirmed that the six main contigs largely represent the complete chromosomes. The remaining contigs include the fully assembled mitochondrial DNA, the linear extrachromosomal DNA harboring ribosomal DNA, and DNA belonging to the *Dictyostelium* Intermediate Repeat Sequence 1 (DIRS1) retrotransposon^26,27^. By performing transcriptomics at three different stages during the lifecycle of *D. firmibasis*, we could annotate 11044 genes (Fig. 1). No haplotypes were detected in the assembly, strongly indicating that *D. firmibasis* is haploid. This is in agreement with the haploid genotype of *D. discoideum*^16^.

## Methods

### Amoebae single cell selection and growth

*D. firmibasis* TNS-C-14 was obtained from the Dicty Stock Centre^25^ (DSC; Strain ID DBS0235812). Approximately 20 cells were scraped from a plaque and resuspended in 250 µl *Escherichia coli* 281 culture grown at 200 rpm, 37 °C, O/N (DSC Strain ID DBS0305927). The bacteria-amoeba mix was plated on 96 mm SM Agar/5 (Formedium) and grown at 22 °C for 3 days. *D. firmibasis* cells were collected from the rim of the plaques formed and were verified by amplifying and sequencing the 18 S ribosomal RNA (rRNA) gene with primers 5′-GTTTGGCCTACCATGGTTGTAA-3′ and 5′-CACCTCTCGCCCCAATATGA-3′. Genomic DNA, used as template, was isolated as described previously^28^ but with Triton-X100 (Sigma-Aldrich) instead of NP40. The sequenced PCR-product was aligned to the *D. firmibasis* TNS-C-14 18 S rRNA gene reference (GenBank: AM168041.1).

For genomic DNA isolation for long- and short-read sequencing, approximately 10^5^ cells originating from a validated plaque, were resuspended in 750 µl *E. coli* 281 O/N culture as described above, plated on three SM Agar/5 plates and grown for 30 h until the majority of bacteria had been consumed and *D. firmibasis* started streaming together to form aggregates. Amoebae from the three plates were harvested using Nunc Cell Scrapers (Thermo Fisher) into 50 ml PDF buffer (20 mM KCl, 9.2 mM K_2_HPO4, 13 mM KH_2_PO_4_, 1 mM CaCl_2_, 2.5 mM MgSO_4_). Cells were harvested at 400xg for 5 min, and washed five times with 50 ml PDF buffer to reduce the number of bacteria.

For mRNA-seq, both *D. firmibasis* and the *D. discoideum* AX2-RRK strain (DSC Strain ID DBS0235521) were grown as above on three SM Agar/5 plates with *E. coli* 281, however after harvesting and washing, one third of the vegetative stage cells were resuspended in 1 ml TRIzol Reagent (Invitrogen) and stored at −20 °C until further processing. One third of the cells was plated onto NN-Agar [8.8 mM KH_2_PO_4_, 2.7 mM Na_2_HPO_4_, 15 g/L Agar] and developed until the slug stage. The remaining third of the cells was plated onto NN-Agar and developed until the fruiting body stage. The developed cells were harvested using Nunc Cell Scrapers, resuspended into 1 ml TRIzol Reagent and stored at −20 °C until further processing. Three biological replicates were prepared for each stage, for a total of 9 mRNA-seq libraries per strain.

### Genomic DNA preparation and sequencing

Genomic DNA (gDNA) was isolated using the Genomic-tip 100/G columns (Qiagen) according to “Protocol: Preparation of Cell Culture Samples” from the Qiagen Genomic DNA Handbook, with the exception of 0.2 mg/ml RNase A (Thermo Scientific) added during lysis of the nuclei in the supplied G2 buffer. 6*10^8^ cells were used as input, which yielded 18.6 µg of gDNA. For long-read sequencing, an initial clean-up was performed by adding 250 µl of *D. firmibasis* genomic DNA (9.3 µg) to 112.5 µl AMPure XP Beads (Beckman Coulter) giving a ratio of 1:0.45 (V/V), to select for high molecular weight DNA. The DNA was bound to the magnetic beads by rotating end-over-end for 5 min, washed twice with 200 µl 70% EtOH and eluted in 62 µl H_2_O by incubating at 37 °C for 10 min, after which the beads were magnetically removed. Further selection of high molecular weight DNA was performed using the Short Read Eliminator (SRE) kit (PacBio) according to the manufacturer’s guide, which yielded 3.2 µg of high molecular weight DNA. Long-read sequencing libraries were prepared from 1.5 µg of gDNA with the SQK-LSK112 Ligation Sequencing Kit (Oxford Nanopore) according to manufacturer’s protocol. 100 ng of the resulting library was sequenced on an R10.4 flowcell (Oxford Nanopore) on the MinION Mk1C (Oxford Nanopore) for 72 h. Basecalling was performed with Guppy v6.3.2 (Oxford Nanopore) using the r104_e81_sup_g610 model. In total, 7.5 Gbp of long-read sequences were generated (Fig. 1).

For short-read sequencing, 4.5 µg *D. firmibasis* genomic DNA was cleaned with AMPure XP Beads as described above but at a 1:0.5 (V/V) ratio. The sequencing library was prepared with 1 µg of DNA using the TruSeq PCR-free DNA sample preparation kit (cat# 20015962, Illumina), according to the TruSeq DNA PCR-free Reference Guide, using unique dual indexes (cat# 20022370, Illumina) and targeting an insert size of 350 bp. The library was sequenced on a NovaSeq. 6000 system (Illumina), with paired-end 150 bp read lengths, on an SP flowcell using v1.5 sequencing chemistry, which resulted in 24.9 Gbp of short-read sequences (Fig. 1). Library preparation and short-read sequencing were performed at SciLifeLab Uppsala.

### mRNA preparation and sequencing

RNA was isolated from three developmental stages of *D. firmibasis* and *D. discoideum*, using TRIzol Reagent (Invitrogen), according to the User Guide, with an additional 75% EtOH wash of the RNA pellet. 4 µg of RNA was DNase treated using DNase I, RNase-free (Thermo Scientific) according to manufacturer’s protocol. The RNA was purified by mixing equal volumes of RNA and Phenol:Chlorofrom:Isoamyl alcohol 25:24:1 (PanReac AppliChem) by vigorous shaking for 15 s, incubating 3 min at 21 °C, followed by centrifuging at 12,000xg for 15 min at 4 °C. The upper aqueous phase was added to three volumes 99% EtOH, 0.1 volumes 3 M NaOAc, incubated on ice for 90 min and centrifuged at 12,000xg for 30 min at 4 °C. EtOH was discarded and the RNA pellet was washed with five volumes 70% EtOH with centrifugation at 12,000xg for 15 min at 4 °C whereafter the pellet was airdried for 2 min, and dissolved in H_2_O.

mRNA sequencing libraries were prepared from 500 ng total RNA using the TruSeq stranded mRNA library preparation kit (cat# 20020595, Illumina) according to manufacturer’s protocol, including polyA selection. Unique dual indexes (cat# 20022371, Illumina) were used. The libraries were sequenced on a NovaSeq 6000 system (Illumina), with paired-end 150 bp read lengths, on an SP flowcell using v1.5 sequencing chemistry. mRNA library preparation and sequencing were performed at SciLifeLab Uppsala.

### Genome assembly and polishing

Adapter trimming and read-splitting of long-reads was performed using Guppy v6.3.2 (Oxford Nanopore). Reads below 1000 bp were filtered out and the best 5 Gbp of data with emphasis on length were kept, using Filtlong v0.2.1 (https://github.com/rrwick/Filtlong). The initial assembly was de-novo assembled from the filtered long-reads using Flye v2.9.1 in nano-hq mode^29^. The assembly was polished in two rounds with the long-reads using Medaka v1.7.2 (Oxford Nanopore). Contigs with a coverage below 50x and contigs of bacterial origin, identified with blastx v2.14.0^30^ to the Swiss-Prot database (release-2022_05)^31^, were discarded. The assembly was polished with NextPolish^32^ using both short- and long-reads with “task = best”, which runs a total of two rounds of polishing with long-reads, followed by four rounds of polishing with short-reads in two different algorithm modes.

In order to calculate the coverage over the genome, the long and short gDNA reads were mapped with minimap2 v2.18^33,34^ using the “-ax map-ont” and “-ax sr” options for the long and short reads, respectively. Coverage over the genome was calculated using samtools v1.14^35^, with the “samtools depth -aa” function, and the average depth was calculated with the avg_depth.py custom python script^36^. The mean long-read coverage over all positions on the 12 *D. firmibasis* contigs was 199x, the mean short-read coverage was 540x, for a total of 739x coverage. NextPolish polishing resulted in 42,885 edits on the 31.5 Mbp assembly (Fig. 1).

**Fig. 2:**
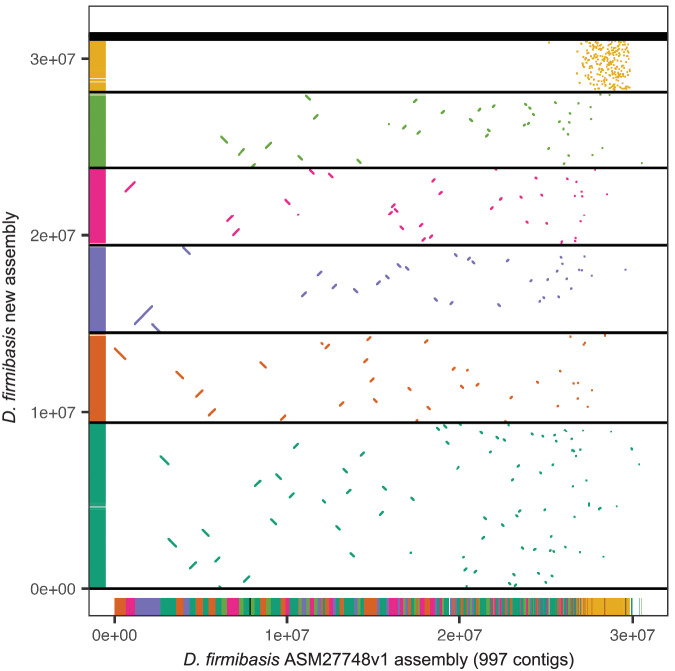
Comparison of the old and new *D. firmibasis* assembly. Regions of the *D. firmibasis* ASM27748v1 assembly^17^ with homology to the new assembly plotted, with a distinct color for each of the large contigs in the new assembly. Matches are plotted in 2D-space and on the x- and y-axes (old and new assembly respectively). Horizontal black lines indicate contig boundaries of the new assembly.

The new assembly was compared to the old *D. firmibasis* ASM27748v1 assembly (GenBank GCA_000277485.1)^17^ using satsuma2 v2016-12-07^37,38^ to identify homologous regions, and with the analyze_genomes.py custom python script^36^ to calculate genome metrics. Synteny analysis revealed that, although the old assembly is highly fragmented, there are no major regions of the new assembly entirely missing in the old assembly (Fig. 2). The two assemblies are also similar in size. The main difference between the assemblies is that the new assembly contains no undetermined bases (versus 4.1 Mbp undetermined in the old genome) and the new genome is much more contiguous, resulting in an assembly more representative of the *D. firmibasis* genome (Table 1). Additionally, the new assembly is more complete, as calculated with BUSCO v5.3.1 based on the Eukaryota Odb10 dataset^39^ (Table 1).

**Table 1:** Old and new *D. firmibasis* assembly statistics.

	Old	New
Genome size (Mbp)	30.6	31.5
Determined bases (Mbp)	26.4	31.5
Undetermined bases	4,112,630	0
# Gaps >10 bp	6312	0
# Contigs	997	12
N50 (Mbp)	0.18	4.39
A + T%	75.2	76.0
BUSCO complete%^39^	82.8	92.9

### Genome annotation

In order to predict and annotate protein coding genes, we made use of both expressed transcript evidence and homology evidence to the known *D. discoideum* proteome. mRNA-seq reads were mapped to the genome using STAR v2.7.10b^40^, and their coverage was calculated as for the gDNA reads above, resulting in a total of 1576x coverage. The mapped mRNA reads were assembled to transcripts using genome-guided Trinity v2.14.0^41^, with a maximum intron size of 5000 bp. The assembly was annotated with MAKER v3.01.04^42^ with the assembled transcripts and the *D. discoideum* UP000002195 UniProt reference proteome^31^ as EST and protein homology evidence, respectively. Homology of annotated *D. firmibasis* proteins to proteins in the *D. discoideum* proteome was identified with blastp v2.14.0^30^.

In total, 379 tRNAs were annotated with tRNAscan-SE v2.0.12^43^, which in line with the number of tRNAs annotated in *D. discoideum* (Table 2). miRNAs were annotated with ShortStack v4.0.1^44^ based on the mature miRNAs reported previously^19^, and *D. firmibasis* small RNA-seq reads from NCBI BioProject PRJNA972620^45^. Class I RNAs were annotated based on sequence, structure and presence of up-stream potential promotor motifs as described previously^10^ (Table 2). Other non-coding RNAs, such as rRNAs, snRNAs and snoRNAs, were annotated using infernal v1.1.4^46^ with a covariance model from Rfam^47^ filtered to Amoebozoa. Transposable elements were identified with tblastx v2.14.0^30^ with e-values below 10^−15^ and reference transposable element sequences from Repbase (Genetic Information Research Institute) filtered to *D. discoideum*.

**Table 2:** Number of annotations in *D. firmibasis* and *D. discoideum*.

	*D. firmibasis*	*D. discoideum*
Protein coding genes	10,564	11,326 (dictyBase curated^25^)
tRNAs	379	418^16^
miRNAs	9	28^11,12,14,59^
Class I RNAs	10	38^13^
snRNAs	28	17^15,60^
snoRNAs	23	47^15,61^

**Fig. 3:**
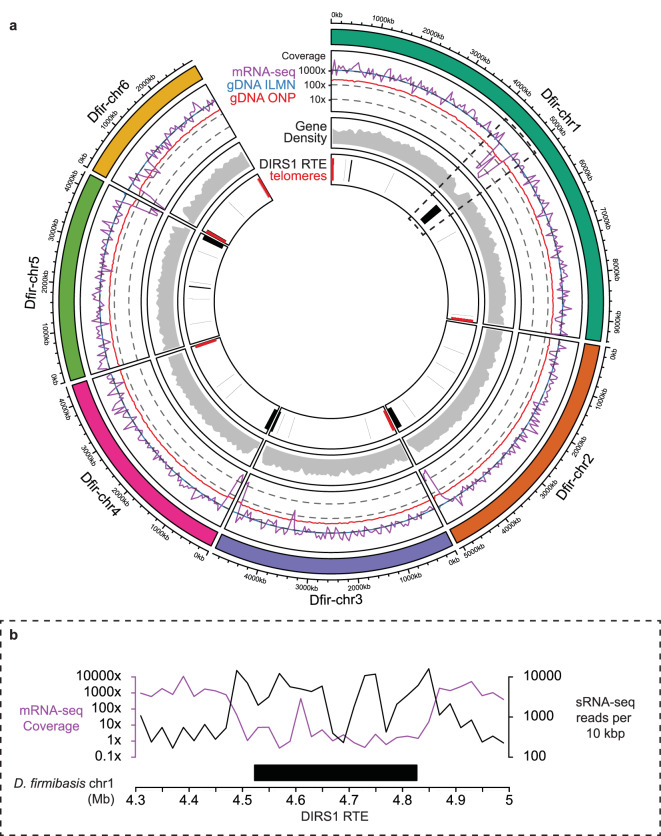
Coverage and annotation of the new *D. firmibasis* assembly. (**a**) Circular representation with the six chromosomal contigs (chr) in the new *D. firmibasis* assembly. From outside to inside: coverage of mRNA-seq (purple line), gDNA from Illumina short-read sequencing (gDNA ILMN, blue line) and gDNA from Oxford Nanopore long-read sequencing gDNA (gDNA ONP, red line); gene density (grey); annotation of DIRS1 retrotransposable elements (DIRS1 RTE, black rectangles) and telomeric repeats (red rectangles). Circular plot generated with the R package *circlize*^62^. (**b**) Close-up of the *D. firmibasis* chr1 contig from position 4.3 Mbp to 5 Mbp, containing a DIRS1 retrotransposable element (DIRS1 RTE, solid black bar), with coverage from mRNA-seq (purple line) and number of small RNA-seq (black line, sRNA-seq) reads mapping per 10 kb region. sRNA-seq reads accessed with NCBI Bioproject accession PRJNA972620^45^. Visualization was performed with the R package karyoploteR v1.28.0^63^.

The combined number of protein coding genes and (nc)RNA genes amounted to a total number of 11044 annotated genes. Gene annotations were manually verified and adjusted where necessary, using NCBI Genome Workbench v3.8.2^48^. The annotated genome has been submitted to NCBI with genome accession number JAVFKY000000000^49^. The combined annotation resulted in a mean gene density of 70.6% which is distributed homogeneously over the main contigs, but reduced towards the ends of the contigs (Fig. 3a). To visualize the coverage of the different sequencing data over the genome, we calculated the mean coverage over 2.5 kbp regular intervals using the custom cov_per_region.py python script^36^. As expected, the coverage of the gDNA reads was constant over the genome, whereas the mRNA-seq coverage was more variable (Fig. 3a). In particular, the mRNA-seq coverage was lower between position 4.5 Mbp and 4.8 Mbp of chr1. This region, which also features relatively low gene density, is covered by DIRS1 elements (Fig. 3a). DIRS1 is known to be targeted by small interfering RNAs in *D. discoideum*^14,50^. Small RNA data from *D. firmibasis*^19,45^ were mapped to the genome using ShortStack v4.0.1^44^, and the number of mapped sRNA reads were calculated over 10 kbp intervals with featureCounts v2.0.3^51^. Coverage of small RNAs was substantially higher on the region featuring DIRS1 than the surrounding regions. This is in agreement with the situation in *D. discoideum* and could be partly responsible for the low mRNA-seq coverage in the region (Fig. 3b). Other large DIRS1 insertions were found at the ends of chromosomes 2, 3, 4, and 5 (Fig. 3a). This is similar to what has been reported for *D. discoideum*, which contains clusters of DIRS1 repeats near one end of each chromosome^16^. When the *D. discoideum* genome was sequenced, no conventional telomeric repeats could be found at the ends of the six chromosomes^16^. In the new *D. firmibasis* assembly however, potential telomeric repeats with sequences 5′-GAGGAGAGAGTCCCTTTTTTT-3′ and 5′-GGGGAGAGACA-3′ could be identified. The repeats were annotated using bowtie v1.3.1^52^ allowing two mismatches in repeat 5′-GAGGAGAGAGTCCCTTTTTTT-3′ and one mismatch per 5′-GGGGAGAGACAGGGGAGAGACA -3′ double repeat. Telomeric repeats were found at both ends of chromosomes 1 and 6, and one end of chromosomes 3 and 4 (Fig. 3a), and were not present elsewhere on the chromosomes. Both types of telomeric repeats could be identified on the same chromosome end, and an average of 15 repeats could be identified per end.

## Data Records

This Whole Genome Shotgun project has been deposited at DDBJ/ENA/GenBank under the accession JAVFKY000000000^49^. The version described in this paper is version JAVFKY010000000. The gDNA and mRNA sequencing libraries have been submitted to SRA with BioProject accession number PRJNA1008051^53^. In total, 18 paired-end mRNA sequencing libraries are associated with this work – 9 for *D. firmibasis* and 9 for *D. discoideum* – from three distinct morphological stages: vegetative, slug and fruiting body stages, identifiable with respectively “0 h”, “16 h” and “24 h” in the repository.

## Technical Validation

### Chromosome-level contiguity and completeness

**Fig. 4:**
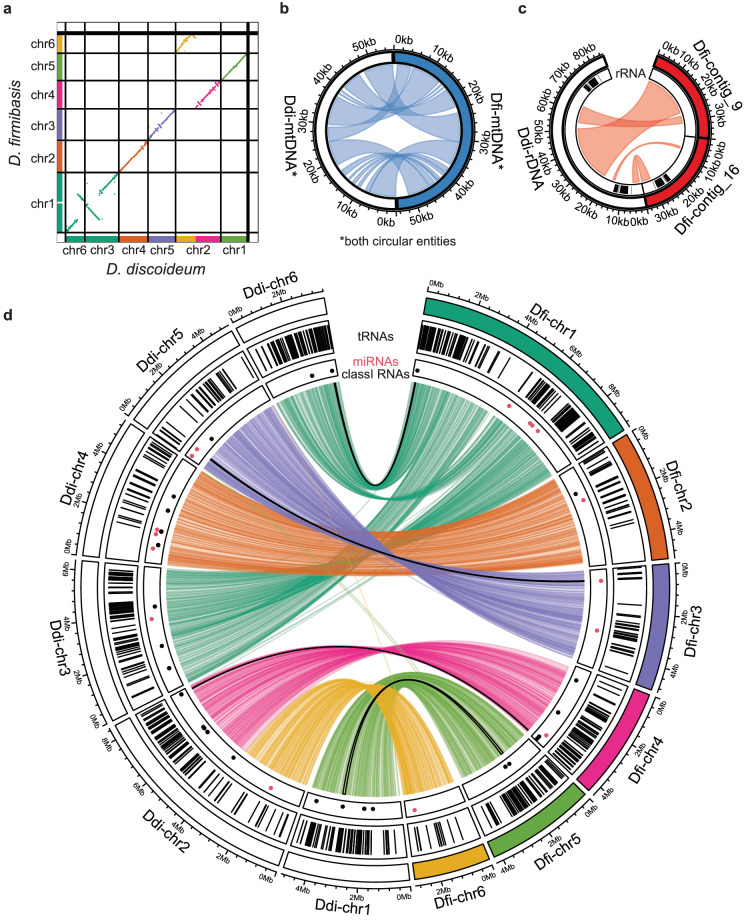
Extensive conservation of synteny between *D. discoideum* AX4 and *D. firmibasis*. (**a**) Comparison of genomes as in Fig. 2 but between the new *D. firmibasis* assembly and the dicty_2.7 *D. discoideum* AX4 assembly, with chromosome numbers indicated. The *D. firmibasis* chromosomes are colored as previously. Regions of the *D. discoideum* dicty_2.7 assembly are colored to match the *D. firmibasis* chromosomes they are homologous to. (**b**) Circular alignment of the mitochondrial genomes from the *D. discoideum* (Ddi-) and *D. firmibasis* (Dfi-) assemblies. Homologous regions are visualized with light blue links. Regions within 500 bp proximity were merged for visual clarity. (**c**) As in (**b**), but for the *D. discoideum* extrachromosomal DNA palindrome containing the ribosomal DNA (rDNA), aligned to *D. firmibasis* contig_9 and contig_16. rDNA locations are represented by black lines in the inner ring. Regions of homology (light red) within 5 kbp proximity in the same direction on both contigs were merged. (**d**) Circular alignment of the chromosomes of the dicty_2.7 *D. discoideum* AX4 assembly and the new *D. firmibasis* assembly. From outside to inside is the visual representation of the contigs, colored for *D. firmibasis*, white for *D. discoideum*; tRNAs on the genome assemblies (black vertical lines); presence of miRNAs (red dots) and Class I RNAs (black dots); and links between the two assemblies to indicate synteny regions based on nucleotide homology. The links are colored according to the *D. firmibasis* contig it matches. Four of the synteny regions contain miRNAs in both assemblies or Class I RNAs in both assemblies – outlined black. Regions of homology within 5 kbp proximity in the same direction on both contigs were merged. Circular plots were generated with the R package *circlize*^62^.

By comparing the new assembly presented here to the first published *D. firmibasis* assembly^17^, we could show that no major parts of the old assembly were missing in the new assembly (Fig. 2a). Similarly, a synteny search was performed between the new *D. firmibasis* assembly and the *D. discoideum* AX4 genome^16,54^ with satsuma2 v2016-12-07^37,38^. *D. discoideum* is relatively closely related to *D. firmibasis*^18^, and in line with that we could identify extensive synteny between the two species (Fig. 4a). There appears to have been no major reorganization of *D. firmibasis* chromosomes 2, 3 and 5, which match *D. discoideum* chromosomes 4, 5 and 1, respectively (Fig. 4a). *D. firmibasis* chromosome 1 matches *D. discoideum* chromosomes 3 and 6, with large inversions. The region of *D. firmibasis* chromosome 1 that harbors the DIRS1 retrotransposon appears to be missing in *D. discoideum*, revealing that this insertion might have occurred more recently or was lost in *D. discoideum*. Chromosome 2 of *D. discoideum* features a 1.5 Mbp inverted duplication^16^. This can be observed here, since a region of the *D. firmibasis* chromosome 6 is represented twice in *D. discoideum* chromosome 2 (Fig. 4a). Strikingly, it is in this same region that the *D. firmibasis* chromosomes 4 and 6 are split, relative to the larger *D. discoideum* chromosome 2 (Fig. 4a). This is in line with the hypothesis, that the region where the duplication is found, is prone to breakage^16^. It should be noted that in contrast to *D. firmibasis*, *D. discoideum* AX4 has a long history of cultivation in laboratories, and was originally selected for axenic growth. This has resulted in a certain degree of genome rearrangement and mutations^55,56^. Since all *D. discoideum* chromosomes are accounted for in the six large *D. firmibasis* contigs, we conclude the new *D. firmibasis* assembly is complete, and of chromosome-level quality.

Besides the chromosomes, synteny in four of the six remaining small contigs was detected as well. One contig represented the *D. firmibasis* mitochondrial DNA, which was assembled as a circular entity, indicating that the entire mitochondrial genome was covered. Between *D. firmibasis* and *D. discoideum* there appear to have been no major reorganizations in the mitochondrial genomes (Fig. 4b). Furthermore, the linear extrachromosomal DNA containing the rRNA genes in *D. discoideum* matched two smaller *D. firmibasis* contigs, which also contain annotations for the 5S, 5.8S, 18  and 28S rRNA genes (Fig. 4c). In *D. discoideum*, this extrachromosomal DNA is palindromic, with rRNA genes on both sides, but in the combined *D. firmibasis* contigs, only one set of the rRNA genes is assembled. In addition, two small contigs are mainly made up of fragments of the retrotransposon DIRS1^26,27^. The remaining contig contains 34 annotated genes, which all have homologues in *D. discoideum* and/or another closely related dictyostelid, *Dictyostelium purpureum*^16,57,58^. This contig has telomeric repeats at one end, suggesting that it may constitute the end of one of the chromosomes.

Much of the homology between the two genomes was detected due to conservation of protein coding regions. However, we were also interested in understanding to what extent the genes coding for ncRNAs were conserved between the two species. Total number of tRNAs is high in both species, with 379 and 418 annotated tRNA genes in the *D. firmibasis* and *D. discoideum* genome^16^, respectively (Table 2). Not only do their numbers match, they also appear to be located in homologous areas of the genome, as seen from the tRNA density plot (Fig. 4d). Small ncRNAs such as Class I RNAs and miRNAs appear to be much less conserved and rapidly evolving, as previously reported^10,19^ (Table 2). Here, we could only detect four synteny regions which contained Class I RNAs or miRNAs in both species (outlined with black lines connecting the genomes in Fig. 4d). Besides these, the majority of Class I RNAs and miRNAs appear to be unique to each species.

### Validation of gene annotation and expression

*D. firmibasis* and *D. discoideum* mRNA-seq reads from three distinct morphological stages (Fig. 1; three biological replicates from each stage) were mapped to their respective genomes using STAR v2.7.10b^40^. Reads were assigned to genes with featureCounts v2.0.3^51^. Using the new *D. firmibasis* assembly, 95% of all reads could be mapped to the genome, and 94% of all mapped reads were assigned to genes, demonstrating completeness of the genome and annotations. To determine the number of annotated genes that was clearly expressed, we defined genes with fewer than 100 mapped reads as not/lowly expressed. Using this cutoff, we detected expression of 94% of the of the annotated genes, i.e. 9934 out of the 10564 annotated protein coding genes. Homology evidence to *D. discoideum* genes could be identified for 10196 of *D. firmibasis* protein coding genes with blastp v2.14.0^30^.

## Data Availability

Software was run with standard parameters unless otherwise indicated. Custom code and scripts, to analyze the genome, annotations, or generate figures is available on GitHub (https://github.com/Bart-Edelbroek/firmibasis_genome) and also on figshare^36^.
